# Baseline Clinical Characterization of Participants in the Accelerating Medicines Partnership Schizophrenia Program

**DOI:** 10.1093/schizbullopen/sgaf012

**Published:** 2025-08-25

**Authors:** Jean Addington, Lu Liu, Monica Chu, Karl Jungert, Nora Penzel, Ofer Pasternak, Emily Farina, Ricardo E Carrion, Cheryl M Corcoran, Vijay A Mittal, Gregory P Strauss, Alison R Yung, Luis Alameda, Celso Arango, Owen Borders, Sylvain Bouix, Nicholas J K Breitborde, Matthew R Broome, Kristin S Cadenhead, Rolando I Castillo-Passi, Eric Yu Hai Chen, Jimmy Choi, Michael J Coleman, Philippe Conus, Covadonga M Diaz-Caneja, Lauren M Ellman, Paolo Fusar Poli, Pablo A Gaspar, Carla Gerber, Louise Birkedal Glenthøj, Leslie E Horton, Christy Lai Ming Hui, Joseph Kambeitz, Lana Kambeitz-Ilankovic, Tina Kapur, Sinead Kelly, Melissa J Kerr, Matcheri S Keshavan, Minah Kim, Sung-Wan Kim, Nikolaos Koutsouleris, Jun Soo Kwon, Kerstin Langbein, Kathryn E Lewandowski, Daniel Mamah, Patricia J Marcy, Daniel H Mathalon, Catalina Mourgues, Merete Nordentoft, Angela R Nunez, Godfrey D Pearlson, Jesus Perez, Diana O Perkins, Albert R Powers, Jack Rogers, Fred W Sabb, Jason Schiffman, Johanna Seitz-Holland, Jai L Shah, Steven M Silverstein, Stefan Smesny, William S Stone, Judy L Thompson, Rachel Upthegrove, Swapna Verma, Jijun Wang, Daniel H Wolf, Tianhong Zhang, Lauren Addamo, Kate Buccilli, Dominic Dwyer, Sophie Todd, Youngsun T Cho, Clara Fontenau, Zailyn Tamayo, Carrie E Bearden, John M Kane, Patrick D McGorry, Rene S Kahn, Martha E Shenton, Scott W Woods, Accelerating Medicines Partnership® Schizophrenia Nelson Barnaby, Monica E Calkins

**Affiliations:** Department of Psychiatry, Hotchkiss Brain Institute, University of Calgary, Calgary, AB T2N 4Z6, Canada; Department of Psychiatry, Hotchkiss Brain Institute, University of Calgary, Calgary, AB T2N 4Z6, Canada; Department of Psychiatry, Hotchkiss Brain Institute, University of Calgary, Calgary, AB T2N 4Z6, Canada; Department of Psychiatry, Hotchkiss Brain Institute, University of Calgary, Calgary, AB T2N 4Z6, Canada; Department of Psychiatry, Massachusetts General Hospital and Harvard Medical School, Boston, MA 02115, United States; Department of Psychiatry, Brigham and Women’s Hospital, Harvard Medical School, Boston, MA 02115, United States; Department of Psychiatry and Psychotherapy, Ludwig-Maximilian-University, 80336 Munich, Germany; Department of Psychiatry, Massachusetts General Hospital and Harvard Medical School, Boston, MA 02115, United States; Department of Psychiatry, Brigham and Women’s Hospital, Harvard Medical School, Boston, MA 02115, United States; Department of Radiology, Brigham and Women’s Hospital and Harvard Medical School, Boston, MA 02115, United States; Department of Psychiatry, Yale University School of Medicine, New Haven, CT 06519, United States; Northwell, Glen Oaks, NY 11004, United States; Donald and Barbara Zucker School of Medicine at Hofstra/Northwell, Hempstead, NY 11549, United States; Institute of Behavioral Science, Feinstein Institutes of Medical Research, Manhasset, NY 11030, United States; Department of Psychiatry, Icahn School of Medicine at Mount Sinai, New York, NY 10029, United States; Department of Psychology, Northwestern University, Evanston, IL 60201, United States; Department of Psychology, University of Georgia, Athens, GA 30602, United States; Institute of Mental and Physical Health and Clinical Translation (IMPACT), Deakin University, Geelong, VIC, 3220, Australia; School of Health Sciences, University of Manchester, Manchester, M139PL, United Kingdom; National Psychosis Unit, South London and Maudsley NHS Foundation Trust, London, SE5 8AF, United Kingdom; Service of General Psychiatry, Treatment and Early Intervention in Psychosis Program, Lausanne University Hospital (CHUV), 1003, Lausanne, Switzerland; Department of Psychosis Studies, Institute of Psychiatry, Psychology and Neuroscience, King’s College, London, SE5 8AF, United Kingdom; Department of Child and Adolescent Psychiatry, Institute of Psychiatry and Mental Health, Hospital General Universitario Gregorio Marañón, IiSGM, CIBERSAM, Instituto de Salud Carlos III, School of Medicine, Universidad Complutense, 28009 Madrid, Spain; Department of Psychiatry, Brigham and Women’s Hospital, Harvard Medical School, Boston, MA 02115, United States; Department of Software Engineering and Information Technology, École de Technologie Supérieure, Montréal, QC HC3 1K3, Canada; Department of Psychiatry and Behavioral Health, Early Psychosis Intervention Center (EPICENTER), College of Medicine, The Ohio State University Wexner Medical Center, Columbus, OH 43210, United States; Institute for Mental Health, University of Birmingham, Birmingham, B15 2TT, United Kingdom; Early Intervention for Psychosis Services, Birmingham Women’s and Children’s NHS Foundation Trust, Birmingham, B13 8QE, United Kingdom; University of California, San Diego, La Jolla, CA 92104, United States; Department of Psychiatry, University of Chile, 833011 Santiago, Chile; Department of Neurology and Psychiatry, Clínica Alemana - Universidad del Desarrollo, 7610658 Santiago, Chile; Department of Psychiatry, School of Clinical Medicine, LKF Faculty of Medicine, University of Hong Kong, Hong Kong SAR, China; Olin Neuropsychiatry Research Center, Hartford HealthCare Behavioral Health Network, Hartford, CT 06106, United States; Department of Psychiatry, Brigham and Women’s Hospital, Harvard Medical School, Boston, MA 02115, United States; General Psychiatry Service, Treatment and Early Intervention in Psychosis Program (TIPP–Lausanne), Lausanne University Hospital and University of Lausanne, 1003 Lausanne, Switzerland; Department of Child and Adolescent Psychiatry, Institute of Psychiatry and Mental Health, Hospital General Universitario Gregorio Marañón, IiSGM, CIBERSAM, Instituto de Salud Carlos III, School of Medicine, Universidad Complutense, 28009 Madrid, Spain; Department of Psychology & Neuroscience, Temple University, Philadelphia, PA 19122-6085, United States; Department of Psychosis Studies, Institute of Psychiatry, Psychology and Neuroscience, King’s College, London, SE5 8AF, United Kingdom; Department of Brain and Behavioral Sciences, University of Pavia, 27100 Pavia, Italy; Department of Psychiatry, University of Chile, 833011 Santiago, Chile; Prevention Science Institute, University of Oregon, Eugene, OR 97401, United States; Oregon Research Institute, Springfield, OR 97477, United States; VIRTU Research Group, Mental Health Copenhagen, University of Copenhagen, 2900 Copenhagen, Denmark; Department of Psychology, University of Copenhagen, 1353 Copenhagen, Denmark; Department of Psychiatry, University of Pittsburgh School of Medicine, Pittsburgh, PA 15213, United States; Department of Psychiatry, School of Clinical Medicine, LKF Faculty of Medicine, University of Hong Kong, Hong Kong SAR, China; Department of Psychiatry, Faculty of Medicine and University Hospital Cologne, University of Cologne, 50937 Cologne, Germany; Department of Psychiatry, Faculty of Medicine and University Hospital Cologne, University of Cologne, 50937 Cologne, Germany; Faculty of Psychology and Educational Sciences, Department of Psychology, Ludwig-Maximilian-University, 80802 Munich, Germany; Department of Radiology, Brigham and Women’s Hospital and Harvard Medical School, Boston, MA 02115, United States; Department of Psychiatry, Brigham and Women’s Hospital, Harvard Medical School, Boston, MA 02115, United States; Orygen, Parkville, VIC, 3052 Australia; Department of Psychiatry, Beth Israel Deaconess Medical Center, Harvard Medical School, Boston, MA 02115, United States; Department of Neuropsychiatry, Seoul National University Hospital, Seoul 03082, South Korea; Department of Psychiatry, Seoul National University College of Medicine, Seoul 03080, South Korea; Department of Psychiatry, Chonnam National University Medical School, Gwangju 61469, South Korea; Mindlink, Gwangju Bukgu Mental Health Center, Gwangju 61005, South Korea; Department of Psychiatry and Psychotherapy, Ludwig-Maximilian-University, 80336 Munich, Germany; Department of Psychosis Studies, Institute of Psychiatry, Psychology and Neuroscience, King’s College, London, SE5 8AF, United Kingdom; Max Planck Institute of Psychiatry, 80804 Munich, Germany; Department of Psychiatry, Seoul National University College of Medicine, Seoul 03080, South Korea; Department of Psychiatry, Hanyang University Hospital, Seoul 04763, South Korea; Department of Psychiatry and Psychotherapy, Jena University Hospital, 07743 Jena, Germany; Psychotic Disorders Division, McLean Hospital, Belmont, MA 02478, United States; Department of Psychiatry, Harvard Medical School, Boston, MA 02115, United States; Department of Psychiatry, Washington University Medical School, St. Louis, MO 63110, United States; Northwell, Glen Oaks, NY 11004, United States; Department of Psychiatry and Behavioral Sciences and Weill Institute for Neurosciences, University of California, San Francisco, San Francisco, CA 94121, United States; Mental Health Service 116D, Veterans Affairs San Francisco Health Care System, San Francisco, CA 94121, United States; Department of Psychiatry, Yale University School of Medicine, New Haven, CT 06519, United States; VIRTU Research Group, Mental Health Copenhagen, University of Copenhagen, 2900 Copenhagen, Denmark; Department of Clinical Medicine, University of Copenhagen, 2200 Copenhagen, Denmark; Department of Psychiatry, Yale University School of Medicine, New Haven, CT 06519, United States; Connecticut Mental Health Center, New Haven, CT 06519, United States; Department of Psychiatry, Yale University School of Medicine, New Haven, CT 06519, United States; Olin Neuropsychiatry Research Center, Hartford Hospital, Hartford, CT 06106, United States; CAMEO, Early Intervention in Psychosis Service, Cambridgeshire and Peterborough NHS Foundation Trust, Cambridge, Cambridgeshire, CB4 1PX, United Kingdom; Department of Medicine, Institute of Biomedical Research (IBSAL), Universidad de Salamanca, 37007 Salamanca, Spain; Department of Psychiatry, University of North Carolina at Chapel Hill, Chapel Hill, NC 27514, United States; Department of Psychiatry, Yale University School of Medicine, New Haven, CT 06519, United States; Connecticut Mental Health Center, New Haven, CT 06519, United States; Institute for Mental Health, University of Birmingham, Birmingham, B15 2TT, United Kingdom; Centre for Human Brain Health, University of Birmingham, Birmingham, B152TT United Kingdom; Prevention Science Institute, University of Oregon, Eugene, OR 97401, United States; Department of Psychological Science, University of California, Irvine, Irvine, CA, United States; Department of Psychiatry, Massachusetts General Hospital and Harvard Medical School, Boston, MA 02115, United States; Department of Psychiatry, Brigham and Women’s Hospital, Harvard Medical School, Boston, MA 02115, United States; Douglas Research Centre, Montréal, QC H4H 1R3, Canada; Department of Psychiatry, McGill University, Montréal, QC H3A 1A1, Canada; Department of Psychiatry, University of Rochester Medical Center, Rochester, NY 14642, United States; Department of Psychiatry and Psychotherapy, Jena University Hospital, 07743 Jena, Germany; Department of Psychiatry, Beth Israel Deaconess Medical Center, Massachusetts Mental Health Center and Harvard Medical School, Boston, MA 02115, United States; Departments of Psychiatry and Neuroscience, University of Rochester Medical Center, Rochester, NY 14642, United States; Institute for Mental Health, University of Birmingham, Birmingham, B15 2TT, United Kingdom; Department of Psychiatry, University of Oxford, Oxford, 0X37JX United Kingdom; Institute of Mental Health, 539747, Singapore; Duke-NUS Medical School, 169857, Singapore; Shanghai Mental Health Center, Shanghai Jiaotong University School of Medicine, Shanghai 20030, China; Department of Psychiatry, Perelman School of Medicine, University of Pennsylvania, Philadelphia, PA 19063, United States; Department of Psychiatry, Shanghai Mental Health Center, Shanghai Jiaotong University School of Medicine, Shanghai 200030, China; Orygen, Parkville, VIC, 3052 Australia; Centre for Youth Mental Health, The University of Melbourne, Parkville, VIC, 3052, Australia; Orygen, Parkville, VIC, 3052 Australia; Centre for Youth Mental Health, The University of Melbourne, Parkville, VIC, 3052, Australia; Orygen, Parkville, VIC, 3052 Australia; Centre for Youth Mental Health, The University of Melbourne, Parkville, VIC, 3052, Australia; Orygen, Parkville, VIC, 3052 Australia; Centre for Youth Mental Health, The University of Melbourne, Parkville, VIC, 3052, Australia; Department of Psychiatry, Yale University School of Medicine, New Haven, CT 06519, United States; Department of Psychiatry, Yale University School of Medicine, New Haven, CT 06519, United States; Department of Psychiatry, Yale University School of Medicine, New Haven, CT 06519, United States; Departments of Psychiatry and Biobehavioral Sciences and Psychology, Semel Institute for Neuroscience and Human Behavior; University of California, Los Angeles, Los Angeles, CA 90095, United States; Department of Psychiatry, Donald and Barbara Zucker School of Medicine, Hempstead, NY 11549, United States; Feinstein Institute for Medical Research, Manhasset, NY 11030, United States; Orygen, Parkville, VIC, 3052 Australia; Centre for Youth Mental Health, The University of Melbourne, Parkville, VIC, 3052, Australia; Department of Psychiatry, Icahn School of Medicine at Mount Sinai, New York, NY 10029, United States; Department of Psychiatry, Massachusetts General Hospital and Harvard Medical School, Boston, MA 02115, United States; Department of Psychiatry and Radiology, Brigham and Women’s Hospital and Harvard Medical School, Boston, MA 02115, United States; Department of Psychiatry, Yale University School of Medicine, New Haven, CT 06519, United States; Orygen, Parkville, VIC, 3052 Australia; Centre for Youth Mental Health, The University of Melbourne, Parkville, VIC, 3052, Australia; Department of Psychiatry, Perelman School of Medicine, University of Pennsylvania, Philadelphia, PA 19063, United States

**Keywords:** clinical high risk, community controls, psychosis, assessments, clinical outcomes, AMP SCZ

## Abstract

**Background:**

This paper focuses on the baseline clinical characterization of the participants in the Accelerating Medicines Partnership Schizophrenia (AMP SCZ) program. The AMP SCZ program is designed to investigate a wide array of clinical variables and biomarkers in a total of 2040 clinical high-risk (CHR) participants and 652 community control (CC) participants.

**Methods:**

The dataset analyzed includes 1642 individuals at clinical high risk for psychosis and 519 CCs. Key measures include the Positive Symptoms and Diagnostic Criteria for the Comprehensive Assessment of At-Risk Mental States Harmonized with the Structured Interview for Psychosis-Risk Syndromes, which determined CHR criteria and the severity of attenuated psychotic symptoms (APS). Other measures included the Structured Clinical Interview for DSM-5, scales to assess negative symptoms, depression, suicidal ideation, substance use, social and role functioning, and a selection of patient-reported outcomes.

**Results:**

CHR participants presented with more severe ratings on all clinical measures and poorer functioning relative to the CC. There were a few significant small associations between measures of APS and other clinical measures.

**Conclusion:**

The results from this study support previous research indicating that CHR individuals face serious clinical challenges beyond the risk of developing psychosis. Findings indicate significant associations among various clinical measures, underscoring the complex nature of the CHR population. Limitations are acknowledged, including the preliminary nature of the data and the need for more in-depth analyses from AMP SCZ papers already in progress. Future work will focus on longitudinal data and further exploration of clinical variables and their relationship with biomarkers.

## Introduction

The Accelerating Medicines Partnership Schizophrenia (AMP SCZ) program consists of 2 large clinical networks: Psychosis-Risk Outcomes Network (ProNET) (28 acquisition sites) and Prediction Scientific Global Consortium (PRESCIENT) (15 acquisition sites); in addition to a research and data center, the Psychosis Risk Evaluation, Data Integration, and Computational Technologies: Data Processing, Analysis, and Coordination Center. The 2 research networks are composed of 43 sites, all enrolling participants, in North and South America, Australia, Europe, and Asia. The AMP SCZ program proposes to study a wide range of clinical and functional outcome variables as well as several biomarkers in 2040 participants at clinical high-risk for developing psychosis (CHR) and 652 community controls (CCs). There are 2 main aims of the AMP SCZ program: the first is the creation of algorithms that will predict the clinical trajectories and outcomes of CHR youth, and the second is to identify tools and outcomes to support the development and testing of new pharmacological treatments for this CHR population.^1^^,^^2^ Comprehensive details about the complete AMP SCZ program can be found at www.ampscz.org*.* Furthermore, a series of open-access papers describing all the modalities under study have recently been published in the journal “Schizophrenia”.^3–8^ At periodic intervals, study data will be shared with the broader research community through the National Institute of Mental Health (NIMH) Data Archive (NDA). Release 3.0 is now available for AMP SCZ investigators, which, for the purpose of this paper, provides approximately 80% of the final baseline clinical data.

It is well established that CHR individuals, in addition to experiencing attenuated psychotic symptoms (APS) typically present with more severe symptoms across other clinical dimensions and poorer functioning than CC. Clinical high-risk as a group presents with increased negative symptoms^9^^,^^10^ relative to control groups. Comorbid diagnoses, particularly depression, are common in CHR youth, and in most samples, about 50% of CHR youth present with a current or past diagnosis of depression as well as more severe ratings on depression scales relative to age-matched controls.^11^^,^^12^ Furthermore, increased rates of suicide, suicide ideation^13–15^ and sleep disturbance,^16^ as well as a link between suicidal ideation and sleep disturbance,^17^ have been observed among CHR individuals. One of the most well-replicated characteristics of CHR as a group is poor social and role functioning.^18–20^ Additionally, there is indication of greater substance use in the CHR group.^21–23^ Other group differences between CHR and CC include increased perceptions of stress^24^ and discrimination.^25^

The aim of this paper is to present the baseline clinical data of AMP SCZ, compare ratings of CHR participants with those of CC, and examine associations amongst the clinical and functioning measures. Such results are important since they (1) can substantiate previous results and further characterize the clinical presentation of this large CHR sample and highlight the areas in which CHR display differences compared to age-matched CC, and (2) demonstrate associations amongst the different variables to help determine potential covariates for the development of future prediction algorithms. In designing the clinical battery, it was important to include a wide range of measures that would serve as outcomes, predictors and covariates.^3^ As a result, there were concerns that such a battery could be burdensome for both evaluators and participants. For efficiency, studies that aim to test new pharmacological treatments will need to be more precise and limited in their selection of clinical measures, choices that can be informed by the results of analyses such as these.

## Methods

Expanded details of the methods of the clinical component of the AMP SCZ program can be found in the clinical methods paper^3^ and on the website www.ampscz.org, under the section “For Scientists” (use direct link www.ampscz.org/scientists/). The standard operating procedures are available and entitled “Clinical Data Acquisition”.

The dataset used in the current analysis was collected and curated by the AMP SCZ Program^2^ and downloaded from the NDA (data release 3.0 doi: 10.15154/81xe-k706).

### Participants

Release 3.0 includes 1642 CHR individuals (ProNET = 823, PRESCIENT = 819) and 519 CC individuals (ProNET = 306, PRESCIENT = 213). Since this is an early data release taking place while data collection is still ongoing, data were not available on all measures for all participants.

Several strategies were employed to recruit participants for the project. Many individuals were referred by medical professionals and clinicians from various hospital and community-based mental health practices and programs, as well as private practices. Referrals were also obtained from educational institutions, including schools and post-secondary organizations, and community agencies. Additionally, participants were referred by family members or self-referred in response to community outreach initiatives, which included targeted advertising on social and mainstream media. Further sources included other studies, general population screenings, and consumer organizations. Community controls were specifically recruited as control participants.

The initial screening assessment determined whether an individual met criteria for the study. There are 2 participant groups in this project: 1 meeting CHR criteria and the other are the CC. Inclusion and exclusion criteria are presented in Table 1.

**Table 1:** Inclusion and Exclusion Criteria

**Inclusion criteria** *CHR and CC inclusion criteria*: (1) Aged 12-30 years inclusive, (2) Ability to give informed consent (parental/guardian consent obtained for participants aged <18 years). *CHR only inclusion criteria:* (3) CAARMS-defined (Trait Vulnerability; Attenuated Psychotic Symptoms; Brief Limited Intermittent Psychotic Symptoms) or Structured Interview for Psychosis-Risk Syndromes-defined (Brief Intermittent Psychotic Syndrome Current Progression; Attenuated Psychotic Symptom Syndrome Current Progression; Genetic Risk and Deterioration Current Progression) diagnostic criteria for CHR, determined using the PSYCHS.
**Exclusion criteria** *CHR and CC exclusion criteria:* (1) Antipsychotic medication exposure equivalent to a total lifetime haloperidol dose of >50 mg, estimated based on available information, or current antipsychotic medication at time of baseline assessment, (2) Documented history of intellectual disability, (3) Past or current clinically relevant central nervous system disorder, (4) Traumatic brain injury rated 7 or above on the Traumatic Brain Injury screening instrument, or (5) Current or past psychotic disorder. *CC only exclusion criteria:* (6) Meet CHR criteria or have a current or past Cluster A personality disorder, (7) Receiving any current treatment with psychotropic medication, (8) Family history (in first-degree relatives) of psychotic spectrum disorders.

Abbreviations: BLIPS, brief limited intermittent psychotic symptoms; CHR, clinical high-risk; CC, community control; CAARMS, comprehensive assessment of at-risk mental states; PSYCHS, The Positive Symptoms and Diagnostic Criteria for the CAARMS Harmonized with the Structured Interview for Psychosis-risk Syndromes.

Clinical high-risk status is determined by at least one of the following:

(1) Attenuated psychotic symptoms (APS) and/or brief limited positive symptoms assessed using the Positive Symptoms and Diagnostic Criteria for the Comprehensive Assessment of At-Risk Mental States (CAARMS) Harmonized with the Structured Interview for Psychosis-Risk Syndromes (PSYCHS);^26^ (2) trait vulnerability and genetic risk and deterioration assessed using the schizotypal personality disorder section from the Structured Clinical Interview for DSM-5,^27^ the Social and Occupational Functioning Assessment Scale,^28^ and the Family Interview for Genetic Studies.^29^

The study was approved by institutional review boards at all ProNET and PRESCIENT sites. All participants provided written informed consent, including parental consent for participants under 18 years.

### Measures

The key clinical measure in the AMP SCZ Program is the PSYCHS, a semi-structured interview. The PSYCHS operationalize the CHR criteria, as well as the severity and type of APS for both the CAARMS^30^ and the SIPS.^31^ The PSYCHS development is described in 2 papers,^26^^,^^32^ and a recent issue of the journal “Early Intervention in Psychiatry” is dedicated to the development of this measure.^33^ The PSYCHS consist of 15 APS and generates relevant CAARMS and SIPS diagnoses for lifetime, past year and past month, as well as an overall APS severity rating. The 15 symptoms include unusual thoughts and experiences, suspiciousness/paranoia, unusual somatic ideas, ideas of guilt, jealous ideas, unusual religious ideas, erotomanic ideas, grandiosity, 6 perceptual abnormality symptoms (auditory, visual, olfactory, gustatory, tactile, and somatic), and disorganized communication.

The Structured Clinical Interview for DSM-5^27^ (Modules A through E) is used in the AMP SCZ project to assess diagnostic criteria for psychotic, mood, and substance use disorders.

Negative symptoms are assessed with the Negative Symptom Inventory-Psychosis Risk (NSI-PR)^9^ designed specifically for CHR individuals in response to the NIMH MATRICS initiative. Psychometric properties are excellent. The NSI-PR includes scores for 5 domains: avolition, asociality, anhedonia, blunted affect, and alogia.

Level of depression is measured with the Calgary Depression Scale for Schizophrenia (CDSS).^34^ One of the most widely used depression measures in clinical trials for psychosis, the CDSS, has been validated for CHR participants.^35^

Suicide attempts and suicidal ideation are assessed with the Columbia-Suicide Severity Rating Scale (C-SSRS). The C-SSRS has excellent psychometric properties for both adolescent and adult populations.^36^ For this paper, we considered only whether participants had made any suicide attempts in their lifetime and the *wish to be dead* and *non-specific suicidal thoughts* in the past month and for the lifetime.

The Brief Psychiatric Rating Scale (BPRS)^37^ is administered as a measure of general psychopathology and has acceptable psychometric properties.^38^^,^^39^ The BPRS provides an overall score on general psychopathology plus subscale scores for positive symptoms, negative symptoms, affective disturbance, disorganization, and activation based on a recent factor analysis.^40^

The primary measures of functioning are the Global Functioning (GF): Social and Role Scales developed by Cornblatt et al. to measure changes in functioning across time in CHR participants.^41^^,^^42^ The GF: Social scale rates peer relationships, conflict, and family involvement. The GF: Role scale assesses performance, setting, and the amount of support needed in school and work.^42^ Each scale ranges from a single score of 1 (extreme dysfunction) to 10 (superior functioning) and generates 3 scores: (1) current level in the past month, (2) highest level in the past year, and (3) lowest level of functioning in the past year prior to the assessment.^41^

Finally, several Patient Reported Outcome Measures (PROMs) are used. Severity of symptoms over the past 7 days is assessed with a Patient Global Impression of Severity where 1 = no symptoms, 2 = mild, 3 = moderate, 4 = severe, and 5 = very severe. The Overall Anxiety Severity and Impairment Scale^43^ is a validated and reliable measure of anxiety severity and related impairment. The 8-item Patient-Reported Outcomes Measurement Information System-Sleep Disturbance^44^ is administered to assess participants’ perceptions of their sleep quality, depth, and restoration within the past 7 days. The Perceived Stress Scale^45^ is used to capture the degree to which situations in participants’ lives are perceived as stressful (i.e., subjective experience of stress levels). The Alcohol, Smoking, and Substance Involvement Screening Test (ASSIST),^46^ a widely used measure endorsed by the World Health Organisation, is used to assess drug and alcohol use. A brief version of the Perceived Discrimination Scale^47^ determines whether participants have experienced discrimination in their lifetime.

### Procedures

All raters underwent intensive training and met predetermined reliability standards to be certified on the key clinical measures, including the PSYCHS, GF: Social and Role, NSI-PR, BPRS, CDSS, and SCID. Intraclass correlations (ICCs), based on initial data, demonstrating the inter-rater reliability between trainees and the gold standard for these measures for each network are presented in Table SS1. A 2-way mixed effects model with absolute agreement type, single measures ICC was selected for our multiple-rating, multiple-rater design. All ICCs are in the excellent range (all >0.8).

To ensure CHR participants met inclusion/exclusion criteria, vignettes were written after the screening visit. These vignettes included a written description of the 15 PSYCHS APS symptoms and ratings for the 4 PSYCHS measurement concepts (description, tenacity/source, distress, and interference) for each symptom endorsed.^26^ The vignette is detailed so that other assessors can review and discuss the symptoms and then derive a reliable rating. Vignettes were reviewed on conference calls for a consensus decision on the symptom ratings, the diagnosis, and potential transitions to psychosis.

### Statistical analysis

Between-group comparisons were conducted using independent samples t-tests for continuous variables and chi-square tests for categorical variables. The Mann–Whitney U test was applied when the assumption of normality was violated. For examining the associations among the clinical variables, Spearman’s rank correlation was used when the data were not normally distributed.

A chi-square test of independence with Bonferroni correction was used to examine racial differences between groups. Since the groups differed in age, a 1-way ANCOVA and binary logistic regressions were conducted to assess group differences in clinical measures while controlling for age.

All statistical analyses were conducted using the IBM Statistical Package for the Social Sciences (SPSS) version 25 and Statistical Analysis Software (SAS) version 9.4.

## Results

### Demographic characteristics

Demographic characteristics are presented in Table 2. No significant sex differences were observed; however, both the CHR and CC groups had a higher proportion of females than males. The higher proportion of females was further examined in a comparison between networks and among different regions to see if there were regional differences that accounted for the higher proportion of females. Comparing networks, 57.0% of the ProNET CHR sample and 71.7% of the PRESCIENT CHR sample were female. Examining the CHR participants from different regions across both networks, in North America 56.8% were female, in Australia 75.7%, in Europe 56.7%, in Asia 70.0%, and in Chile, the only South American site, 70.0% were female.

**Table 2:** Sociodemographics at Baseline

	**CHR** ***n* = 1642**		**CCs** ***n* = 519**		**Test statistic**	**Significance value**	**Effect size**
	Mean (SD)		Mean (SD)	*t*		*P*	Cohens *d*
Age	21.2 (4.0)		21.6 (3.6)	−2.24		.025	0.105
Years of education	13.0 (2.8)		13.9 (2.7)	−6.84		<.0001	0.327
	Frequency (%)		Frequency (%)		*X^2^*	*P* value	
Sex							
Male	585	(35.63)	200	(38.54)	1.44	.2298	
Female	1057	(64.37)	319	(61.46)			
Racial^a^							
Indigenous groups	19	(1.20)	4	(0.78)	56.40	<.0001	
Native Hawaiian or Pacific Islander	5	(0.31)	0	(0.00)			
East Asian	139	(8.75)	91	(17.74)			
South Asian	78	(4.91)	49	(9.55)			
Southeast Asian	92	(5.79)	27	(5.26)			
Black	137	(8.63)	32	(6.24)			
West/Central Asian and Middle Eastern	52	(3.27)	8	(1.56)			
White	925	(58.25)	268	(52.24)			
Multiracial	141	(8.88)	34	(6.63)			
Marital status							
Single/never married	1269	(77.81)	382	(73.89)	5.16	.1598	
In a relationship	316	(19.37)	123	(23.79)			
Married/common law	44	(2.70)	11	(2.13)			
Divorced	2	(0.12)	1	(0.19)			
Living arrangements^b^							
With family/spouse	951	(58.38)	254	(49.22)	17.95	.0013	
Independent	622	(38.18)	248	(48.06)			
Supported residential	12	(0.74)	3	(0.58)			
Without housing	8	(0.49)	0	(0.00)			
Other	36	(2.21)	11	(2.13)			

Abbreviations: CHR, clinical high-risk; CC, community control.

a East Asian includes: Chinese, Japanese, Korean; South Asian includes: Cambodian, Indonesian, Vietnamese; Southeast Asian includes: Indian, Pakistani, Sri Lankan; West/Central Asian and Middle Eastern includes: Egyptian, Lebanese, Emiratis, Afghans, Iranian.

b Independent includes living alone, with roommates; Other includes unknown, with another adult or student accommodations.

A small, yet significant, age difference was noted between CHR and CC, with CC being slightly older (Cohen’s *d* = 0.11), along with a small but significant difference in years of education, most likely partly due to age difference and partly due to expected premorbid risks or illness consequences delaying education in a subset of CHR (Cohen’s *d* = 0.32). There was a significant difference in race. Results of a chi-square test of independence with Bonferroni correction indicated significant group differences among white, East Asian, and South Asian participants. A significantly greater proportion of East Asian (χ^2^ = 31.14, Bonferroni-adjusted *P* < .009) and South Asian (χ^2^ = 13.43, Bonferroni-adjusted *P* < .009) participants were in the CC group compared to the CHR group, while a significantly greater proportion of white participants (χ^2^ = 8.105, Bonferroni-adjusted *P* = .036) were in the CHR group. Finally, most likely related to the age difference and possible delay in education as noted above, a higher level of education was achieved by the CC group (χ^2^ = 74.05, *P* < .0001), with more participants achieving post-secondary qualifications. See Table S2 for more details.

### Clinical measures

**Figure 1:**
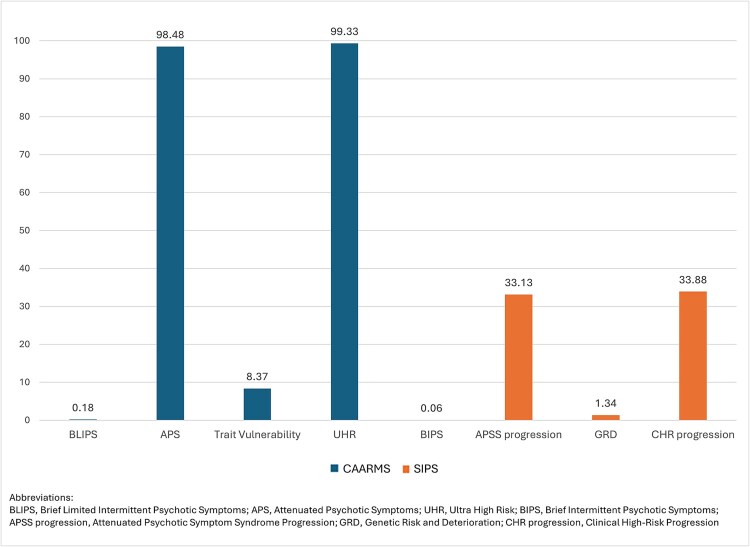
Percentages Meeting Different High-Risk Criteria from the PSYCHS. The first 4 columns refer to the 3 CAARMS Criteria of BLIPS, APS, and Trait Vulnerability, and UHR being the Total Number Meeting at Least One of the 3 CAARMS Criteria. The next 4 columns refer to the 3 SIPS Criteria of BIPS, APSS Progression and GRD, and CHR Progression being the Total Number Meeting at Least One of the 3 SIPS Criteria.

The percentage of participants who met any one of the different diagnostic criteria for the CAARMS (APS, brief limited intermittent psychotic symptoms [BLIPS], trait and vulnerability) and/or for the SIPS (attenuated psychotic symptom syndrome [APSS], brief intermittent psychotic symptoms [BIPS], genetic risk and deterioration [GRD]) are presented in Figure 1. The total percentage meeting CAARMS criteria (ultra high risk [UHR]) and the total percentage meeting SIPS criteria (CHR progression) are also presented in Figure 1. For the CAARMS criteria, 0.18% met BLIPS, 98.48% met APS, and 8.37% met trait vulnerability, with a total of 99.33% of the sample meeting at least one of the CAARMS criteria. For the SIPS, 0.06% met BIPS, 33.13% met APSS progression, and 1.34% met GRD criteria, with a total of 33.88% meeting at least one of the SIPS criteria. These numbers are presented in Figure 1. Note that CHR participants may meet more than 1 criterion.

Table 3 presents the differences between CHR and CC participants. Clinical high-risk participants had more severe ratings on all clinical measures. A significantly higher proportion of CHR participants were diagnosed with major depressive disorder, persistent depressive disorder, bipolar 1, and bipolar 2. No statistically significant differences were found for other depressive disorders, cyclothymia, or other bipolar disorders, likely due to the small sample sizes. Due to age differences, a 1-way ANCOVA was conducted to examine group differences in clinical measures while controlling for age. All outcomes that were statistically significant prior to adjustment remained significant after controlling for age. Additionally, binary logistic regression was used to assess the effects of group and age on the group differences in the C-SSRS and SCID diagnoses. After controlling for age, the group difference in C-SSRS remained significant. For SCID diagnoses, only major depressive disorder without psychosis and persistent depressive disorder remained significant after adjusting for age.

**Table 3:** Comparisons Between CHR Participants and CCs for Clinical Measures

	**CHR** ***n* = 1642**		**CCs** ***n* = 519**		**Test statistic**	**Significance value**	**Effect size**
	*Mean (SD)*		*Mean (SD)*		*t*	*P*	*Cohens d*
PSYCHS total	19.01	(9.34)	2.58	(3.32)	60.18	<.001	1.98
SIPS total	11.27	(4.07)	1.89	(2.16)	67.80	<.001	2.53
CAARMS total	38.15	(17.88)	3.76	(5.13)	68.00	<.001	2.18
NSI-PR							
Anhedonia	1.63	(1.12)	0.80	(0.83)	16.69	<.0001	0.84
Asociality	2.07	(1.07)	0.98	(0.90)	21.20	<.0001	1.10
Avolition	1.76	(1.07)	0.69	(0.70)	24.60	<.0001	1.18
Blunted affect	0.81	(1.03)	0.25	(0.54)	14.65	<.0001	0.68
Alogia	0.58	(0.97)	0.25	(0.63)	8.48	<.0001	0.40
CDSS total	6.28	(4.49)	1.00	(1.78)	36.93	<.0001	1.54
BPRS total	40.92	(8.76)	27.07	(3.66)	47.58	<.0001	2.06
Affective subscore	7.25	(3.18)	3.65	(1.27)	34.95	<.0001	1.48
Positive subscore	6.80	(2.76)	3.14	(0.45)	47.77	<.0001	1.85
Negative subscore	4.34	(2.03)	3.36	(0.90)	14.16	<.0001	0.62
Activation subscore	3.61	(1.27)	3.09	(0.45)	13.06	<.0001	0.54
Disorganization subscore	3.91	(1.35)	3.14	(0.56)	17.21	<.0001	0.74
GF: Social							
Current	7.18	(1.37)	8.51	(0.94)	−23.35	<.0001	1.13
Highest in past year	7.53	(1.26)	8.65	(0.87)	−21.45	<.0001	1.03
Lowest in past year	6.46	(1.42)	8.05	(1.05)	−25.59	<.0001	1.27
Decline in past year	0.34	(0.66)	0.14	(0.43)	7.62	<.0001	0.35
GF: Role							
Current	6.96	(1.94)	8.59	(1.14)	−22.00	<.0001	1.02
Highest in past year	7.51	(1.60)	8.73	(1.06)	−18.74	<.0001	0.89
Lowest in past year	6.24	(2.01)	8.25	(1.25)	−25.38	<.0001	1.20
Decline in past year	0.54	(1.11)	0.14	(0.41)	11.36	<.0001	0.47
	*Frequency (%)*		*Frequency (%)*		*X^2^*	*P value*	
C-SSRS							
Wish to be dead (lifetime)	1168	(81.28)	110	(22.82)	554.48	<.0001	
Wish to be dead (past month)	514	(35.87)	13	(2.73)	196.77	<.0001	
Non-specific suicidal thoughts (lifetime)	941	(65.57)	60	(12.42)	409.15	<.0001	
Non-specific suicidal thoughts (past month)	260	(18.16)	1	(0.21)	97.64	<.0001	
SCID diagnoses							
Depressive disorders							
Major depressive disorder w/o psychosis	484	(49.90)	45	(12.61)	151.38	<.0001	
Persistent depressive disorder	172	(17.75)	2	(0.56)	67.62	<.0001	
Other depressive disorders^a^	16	(1.65)	4	(1.12)	0.49	.4818	
Bipolar disorders							
Bipolar 1 without psychosis	34	(3.51)	0	(0.00)		<.0001^c^	
Bipolar 2	16	(1.65)	0	(0.00)		.0093^c^	
Cyclothymia	3	(0.31)	0	(0.00)		.568^c^	
Other bipolar disorders^b^	8	(0.82)	0	(0.00)		.1175	

Abbreviations: BPRS, brief psychiatric rating scale; CHR, clinical high-risk; CC, community control; C-SSRS, Columbia-suicide severity rating scale; CAARMS, comprehensive assessment of the at-risk mental states; CDSS, Calgary depression scale for schizophrenia; GF: Social, global functioning: Social scale; GF: Role, global functioning: Role scale; NSI-PR, negative symptom inventory—psychosis risk; PSYCHS, positive symptoms and diagnostic criteria for the CAARMS harmonized with the SIPS; SIPS, structured interview for psychosis risk syndromes; SCID, structured clinical interview for DSM-5.

a Other depressive disorders includes substance/medication induced, depression due to another medical condition, and other specified or unspecified depression disorders.

b Other bipolar disorders includes substance/medication induced, due to another medical condition, and other specified or unspecified bipolar disorders.

c Fisher’s exact test was used instead of the Chi-square test.

Compared to CC, CHR participants had higher rates of substance use disorders as well as greater severity. On the ASSIST, except for alcohol, CHR participants were more likely to endorse having used substances compared to CC participants (see Table 4). For PROMs, CHR participants had significantly poorer ratings on all measures. The 1 exception was the PROMIS question about the quality of sleep in the past week, where no difference was observed (see Table 5).

**Table 4:** Comparisons Between CHR Participants and CCs for Substance Use

	**CHR** ***n* = 969**		**CCs** ***n* = 357**		**Test statistic**	**Significance value**	
	Frequency (%)		Frequency (%)		*X* ^2^	*P* value
**DSM -5 substance use diagnoses**						
Alcohol use disorder					34.34	<.0001
Absent	764	(81.19)	321	(94.41)		
Mild	89	(9.46)	10	(2.94)		
Moderate	40	(4.25)	6	(1.76)		
Severe	48	(5.1)	3	(0.88)		
Cannabis use disorder					54.05	<.0001
Absent	727	(77.42)	323	(95.0)		
Mild	80	(8.52)	10	(2.94)		
Moderate	56	(5.96)	5	(1.47)		
Severe	76	(8.09)	2	(0.59)		
Any substance use disorder						
Absent	624	(66.24)	311	(91.47)	82.11	<.0001
Mild	127	(13.48)	16	(4.71)		
Moderate	67	(7.11)	7	(2.06)		
Severe	124	(13.16)	6	(1.76)		
**ASSIST ratings for lifetime use** ^**a**^	**CHR** ***n* = 1508**		**CCs** ***n* = 491**			
Tobacco products	718	(47.68)	161	(32.86)		<.0001
Alcohol	1228	(81.43)	385	(78.41)		0.1477
Cannabis	881	(58.42)	209	(42.57)		<.0001
Cocaine	202	(13.4)	28	(5.70)		<.0001
Amphetamine type stimulants	252	(16.72)	29	(5.91)		<.0001
Inhalants	137	(9.08)	11	(2.24)		<.0001
Sedatives	158	(10.51)	10	(2.04)		<.0001
Hallucinogens	354	(23.47)	51	(10.41)		<.0001
Opioids	84	(5.57)	4	(0.81)		<.0001
Other	101	(6.70)	10	(2.04)		<.0001

Abbreviations: ASSIST, the alcohol, smoking, and substance involvement screening test; CHR, clinical high-risk; CC, community control.

a Fisher’s exact test was used instead of the Chi-square test.

**Table 5:** PROMs: Comparisons Between CHR Participants and CCs

	**CHR**			**CCs**			**Test statistic**	**Significance value**	**Effect size**
	**Mean (SD)**		***N***	**Mean (SD)**		***N***	***t***	***P***	**Cohens *d***
Measures									
PGI-S	2.69	(0.86)	1559	1.32	(0.63)	496	37.90	<.0001	1.90
OASIS total	8.74	(3.98)	1486	3.12	(2.94)	483	33.19	<.0001	1.63
Perceived discrimination total	2.89	(2.11)	1463	1.67	(1.87)	483	11.97	<.0001	0.62
PSS	22.76	(6.54)	1470	12.71	(5.80)	474	31.77	<.0001	1.62
PROMIS: Sleep disturbance score	20.13	(5.74)	1477	14.71	(4.86)	486	20.34	<.0001	1.01
	Median (IQR)		*N*	Median (IQR)		*N*	*Z*	*P*	*r*
PROMIS: Sleep quality score	3	(2)	1479	3	(2)	485	1.46	.1432	0.03

Abbreviations: OASIS, overall anxiety severity and impairment scale; PGI-S, patient global impression of severity; PSS, perceived stress scale; PROMIS, patient reported outcomes measurement information system.

Table 6 presents the correlations between the 3 total scores for APS from the PSYCHS, SIPS, and CAARMS with the other clinical measures for CHR participants. After applying a Bonferroni correction, all significant correlations with *P* < .0001 remained significant. Due to the large sample size, correlations *>*0.8 are considered large, 0.5-0.79 moderate, and 0.2-0.49 small. Correlations below 0.2 are considered to be nonsignificant. Significant moderate and large correlations are bolded. The PSYCHS was significantly correlated with both the SIPS and the CAARMS. Significant correlations between the BPRS positive subscore and the PSYCHS, SIPS, and CAARMS were all moderate correlations, as were the SIPS and the BPRS total score. All remaining significant correlations between the PSYCHS, SIPS, and CAARMS and other clinical measures were small and included the CDSS, depression and disorganization on the BPRS, anxiety, and perceived stress.

**Table 6:** Correlation of Clinical Measures with APS

	**PSYCHS total**	**SIPS total**	**CAARMS total**
	*Spearman ρ*	*Spearman ρ*	*Spearman ρ*
PSYCHS total	1	0.88^****^	0.77^****^
SIPS total	0.88^****^	1	0.81^****^
CAARMS total	0.77^****^	0.81^****^	1
NSI-PR			
Anhedonia	ns	ns	ns
Asociality	ns	ns	0.20^****^
Avolition	ns	ns	0.20^****^
Blunted affect	ns	ns	ns
Alogia			
CDSS total	0.29^****^	0.25^****^	0.29^****^
C-SSRS			
Wish to be dead (lifetime)	ns	ns	ns
Wish to be dead (past month)	0.21^****^	ns	ns
Non-specific suicidal thoughts (lifetime)	ns	ns	ns
Non-specific suicidal thoughts (past month)	ns	ns	ns
BPRS total	0.48^****^	0.50^****^	0.48^****^
Affective subscore	0.29^****^	0.24^****^	0.29^****^
Positive subscore	0.55^****^	0.57^****^	0.52^****^
Negative subscore	ns	ns	ns
Activation subscore	ns	0.20^****^	ns
Disorganization subscore	0.21^****^	0.26^****^	0.23^****^
GF: Social	ns	ns	ns
GF: Role	ns	ns	ns
PGI-S	0.20^****^	ns	0.23^****^
OASIS total	0.26^****^	0.23^****^	0.28^****^
Perceived discrimination total	ns	ns	ns
PSS	0.25^****^	0.22^****^	0.24^****^
PROMIS: Sleep quality score	ns	ns	ns
PROMIS: Sleep disturbance score	ns	ns	ns

^*^ *P* < .05, ^**^P < .01, ^***^P < .001, and ^^****^^P < .0001.

Abbreviations: BPRS, brief psychiatric rating scale; CAARMS, comprehensive assessment of the at-risk mental states; CDSS, Calgary depression scale for schizophrenia; C-SSRS, Columbia-suicide severity rating scale; GF: Social, global functioning: Social scale; GF: Role, global functioning: Role scale; NSI-PR, negative symptom inventory—psychosis risk; OASIS, overall anxiety severity and impairment scale; PSYCHS, positive symptoms and diagnostic criteria for the CAARMS harmonized with the SIPS; SIPS, structured interview for psychosis risk syndromes; PGI-S, patient global impression of severity; PSS, perceived stress scale; PROMIS, patient reported outcomes measurement information system. ns, not significant. Significant moderate and large correlations are bolded.

For readers interested in further details, correlations among the other clinical measures are presented in Table S3a-d.

## Discussion

This paper presents descriptive statistics on the baseline clinical variables from the AMP SCZ program’s third release of data. The clinical component includes a wide range of clinical and functioning measures, as well as a selection of PROMs. Significant differences were observed between the CHR group and the CC on all measures, consistent with previously published results in a range of CHR samples.

The AMP SCZ sample showed higher rates of comorbid diagnoses, particularly major depression, as well as elevated levels of depression and suicidal ideation.^11–15^ Anxiety was also prevalent in the CHR group, consistent with other reports.^48^ Typically CHR individuals exhibit increased negative symptoms compared to healthy control groups,^9^^,^^10^ and in this sample significant differences were observed across all 5 areas of the newly developed NSI-PR. Social and role functioning deficits, widely replicated in previous studies,^18–20^ were again observed here. In terms of substance use, a greater percentage of individuals in the CHR group had a substance use disorder, and except for alcohol use, there was a higher incidence of other substance use among CHR individuals. Although this aligns with other findings,^21–23^ it contrasts with results from the PSYSCAN multi-center study, which did not find substance use differences between healthy controls and CHR, except for a significant difference in favoring controls in alcohol use.^49^ Sleep disturbance remained a concern for CHR individuals in line with earlier research.^16^^,^^17^ Finally, CHR individuals reported higher perceptions of discrimination and stress, corroborating other studies.^24^^,^^25^ These differences demonstrate that CHR individuals often have other serious clinical concerns in addition to being at risk for developing psychosis.

Using the PSYCHS helps to evaluate diagnostic criteria for CHR individuals, producing both CAARMS and SIPS criteria. Few participants (*n* = 4) met BIPS or BLIPS criteria, indicating rarity. Similarly, few participants met trait vulnerability or GRD criteria. Most participants met either APS or APS progression criteria, with nearly 99% meeting CAARMS APS criteria and only 33.1% meeting SIPS APSS criteria. The difference lies in the time frame definition for APS: the SIPS requires a stricter timeline compared to the CAARMS. Almost all the sample met at least 1 CAARMS criterion, while only a third met a SIPS criterion. As the study continues, the impact of meeting SIPS or CAARMS criteria on outcomes will be determined.

Associations among measures of attenuated psychotic symptom severity were significant, which would be expected, as were moderate associations with the BPRS positive symptom subscore. However, associations between attenuated psychotic symptom severity and other clinical measures such as depression, anxiety, and stress were small. These findings underscore the distinction between attenuated positive symptoms and other kinds of symptoms.

The strength of this paper lies in its comprehensive description of the baseline clinical characteristics of a large international sample of CHR and CC participants. This provides a solid foundation for future analyses of the data from the AMP SCZ program and will assist investigators in designing clinical trials. For investigators outside the AMP SCZ program who may have hypotheses they wish to test in the NDA database, this paper offers an overview of basic sample characteristics. A recent special issue of *Schizophrenia Research* highlighted that “embracing heterogeneity creates new opportunities for understanding and treating those at clinical high risk for psychosis.”^50^ The AMP SCZ program has developed a range of measures that can help dissect this heterogeneity and advance progress toward new treatments.

The limitations of this study include that, at this stage, we are only able to report on clinical data from approximately 80% of the expected baseline data on completion of recruitment. It would be valuable to statistically compare these clinical variables with samples from other large consortia, particularly international groups. More detailed analyses of the measures, such as factor and network analyses, would add valuable information. Correlation of these clinical scales with the biomarkers being assessed in AMP SCZ may also help with designing a shorter, potentially more valid, and less burdensome clinical battery. However, progress in the clinical area of AMP SCZ already includes more than 40 approved and in-progress papers on clinical data. These papers address the additional questions that may be raised from this paper, including more complex analyses, comparisons with legacy data, and in-depth examination of individual clinical variables. This does not include several planned papers that will examine both clinical and biomarker data.

In conclusion, CHR individuals face various clinical issues beyond APS that are not necessarily linked to APS. Future work will allow for a more detailed examination of these clinical variables, and with additional data releases, longitudinal data will become available, including information on transitions to psychosis.

## Supplementary Material

Supplementary_Material_revised_sgaf012

## Data Availability

Data presented in this paper are available in the National Institute of Mental Health (NIMH) Data Archive (NDA). On the NDA site (*NIMH Data Archive*—*AMPSCZ*), there is information about available data and how it can be obtained.
