# Correction to: Baseline Clinical Characterization of Participants in the Accelerating Medicines Partnership Schizophrenia Program

**DOI:** 10.1093/schizbullopen/sgag039

**Published:** 2026-10-11

This is a correction to: Jean Addington, Lu Liu, Monica Chu, Karl Jungert, Nora Penzel, Ofer Pasternak, Emily Farina, Ricardo E Carrion, Cheryl M Corcoran, Vijay A Mittal, Gregory P Strauss, Alison R Yung, Luis Alameda, Celso Arango, Owen Borders, Sylvain Bouix, Nicholas J K Breitborde, Matthew R Broome, Kristin S Cadenhead, Rolando I Castillo-Passi, Eric Yu Hai Chen, Jimmy Choi, Michael J Coleman, Philippe Conus, Covadonga M Diaz-Caneja, Lauren M Ellman, Paolo Fusar Poli, Pablo A Gaspar, Carla Gerber, Louise Birkedal Glenthøj, Leslie E Horton, Christy Lai Ming Hui, Joseph Kambeitz, Lana Kambeitz-Ilankovic, Tina Kapur, Sinead Kelly, Melissa J Kerr, Matcheri S Keshavan, Minah Kim, Sung-Wan Kim, Nikolaos Koutsouleris, Jun Soo Kwon, Kerstin Langbein, Kathryn E Lewandowski, Daniel Mamah, Patricia J Marcy, Daniel H Mathalon, Catalina Mourgues, Merete Nordentoft, Angela R Nunez, Godfrey D Pearlson, Jesus Perez, Diana O Perkins, Albert R Powers, Jack Rogers, Fred W Sabb, Jason Schiffman, Johanna Seitz-Holland, Jai L Shah, Steven M Silverstein, Stefan Smesny, William S Stone, Judy L Thompson, Rachel Upthegrove, Swapna Verma, Jijun Wang, Daniel H Wolf, Tianhong Zhang, Lauren Addamo, Kate Buccilli, Dominic Dwyer, Sophie Todd, Youngsun T Cho, Clara Fontenau, Zailyn Tamayo, Carrie E Bearden, John M Kane, Patrick D McGorry, Rene S Kahn, Martha E Shenton, Scott W Woods, Accelerating Medicines Partnership® Schizophrenia Nelson Barnaby, Monica E Calkins, Baseline Clinical Characterization of Participants in the Accelerating Medicines Partnership Schizophrenia Program, *Schizophrenia Bulletin Open*, Volume 6, Issue 1, January 2025, sgaf012, https://doi.org/10.1093/schizbullopen/sgaf012

In the originally published version of this manuscript, author Paolo Fusar-Poli’s name was misspelled.

These details have been corrected only in this correction notice to preserve the published version of record.

