# Supplementary material for: Baseline Clinical Characterization of Participants in the Accelerating Medicines Partnership Schizophrenia Program
Source: Schizophr Bull Open. 2025 Aug 25;6(1):sgaf012. doi: 10.1093/schizbullopen/sgaf012 (PMC12377803; doi:10.1093/schizbullopen/sgaf012)
Supplement: Supplementary_Material_revised_sgaf012 [file supplementary_material_revised_sgaf012.docx]

**SUPPLEMENTARY MATERIAL**

**Supplementary Table 1: Intraclass Correlations between Interviewers and Gold Standards on Key Clinical Measures on Training Case Videos**

| **Measure** | **Total** | **ProNET** | **PRESCIENT** |
| --- | --- | --- | --- |
| ***PSYCHS*** | ***n=116*** | ***n=84*** | ***n=32*** |
| Case 1 - Psychotic | 0.995 | 0.983 | 0.991 |
| Case 2 - Clinical High Risk | 0.965 | 0.964 | 0.967 |
|  | | | |
| ***Negative Symptom Inventory-PR*** | ***n=239*** | ***n=65*** | ***n=174*** |
| Case 1 - moderately severe negative symptoms | 0.986 | 0.986 | 0.986 |
| Case 2 - severe negative symptoms | 0.995 | 0.995 | 0.995 |
|  | | | |
| ***Brief Psychiatric Rating Scale*** | ***n=204*** | ***n=145*** | ***n=59*** |
|  | 0.850 | 0.846 | 0.864 |
|  | | | |
| ***Global Functioning Scales*** | ***n=201*** | ***n=139*** | ***n=62*** |
|  | 0.928 | 0.928 | 0.939 |
|  | | | |
| ***Calgary Depression Scale (CDSS)*** | ***n=221*** | ***n=158*** | ***n=63*** |
|  | 0.816 | 0.808 | 0.843 |
|  | | | |
| ***Structured Clinical Interview for DSM-5*** | ***n=194*** | ***n=145*** | ***n=49*** |
|  | 0.996 | 0.996 | 0.996 |
|  | | | |

*Agreement expressed as ICC values <0.40, poor; 0.40-0.59, fair; 0.60-0.74, good; >0.75, excellent

PSYCHS, Positive Symptoms and Diagnostic Criteria for the CAARMS Harmonized with the SIPS

| **Supplementary Table 2: Maximum Level of Education Achieved** | | | | | | |
| --- | --- | --- | --- | --- | --- | --- |
|  | **CHR**  **n=1629** | | **Community Controls**  **n=518** | | **Test**  **Statistic** | **Significance**  **value** |
|  | *Frequency (%)* | | *Frequency (%)* | | *X^2^* | *p value* |
| Less than seven years | 30 | (1.84) | 8 | (1.54) | 74.05 | <.0001 |
| Junior high school ^a^ | 86 | (5.28) | 17 | (3.28) |  |  |
| Some high school ^b^ | 347 | (21.30) | 52 | (10.04) |  |  |
| Completed high school ^c^ | 444 | (27.26) | 110 | (21.24) |  |  |
| Partial college training | 366 | (22.47) | 144 | (27.80) |  |  |
| College or university education | 294 | (18.05) | 146 | (28.19) |  |  |
| Graduate or professional training | 62 | (3.81) | 41 | (7.29) |  |  |
| ^a^ Junior high school includes up to Grade 9 or a total of nine years of education.  ^b^ Some high school includes nine to eleven years of education.  ^c^ Completed high school includes twelve to thirteen years of education. | | | | | | |

| **Supplementary Table 3a: Correlation of Clinical Measures with Depression and Suicide Measures** | | | | | |
| --- | --- | --- | --- | --- | --- |
|  | **CDSS total** | **C-SSRS: *Wish to be dead (lifetime)*** | **C-SSRS: *Non-specific suicidal thoughts (lifetime)*** | **C-SSRS: *Wish to be dead***  ***(past month)*** | **C-SSRS: *Non-specific suicidal thoughts (past month)*** |
| CDSS total |  | -0.28**** | -0.29**** | **0.50****** | 0.42**** |
| NSI-PR: *Anhedonia* | 0.28**** |  |  |  |  |
| NSI-PR: *Asociality* | 0.23**** |  |  |  |  |
| NSI-PR: *Avolition* | 0.34**** |  |  | 0.22**** |  |
| NSI-PR: *Blunted affect* |  |  |  |  |  |
| NSI-PR: *Alogia* |  |  |  |  |  |
| BPRS: *Activation subscore* |  |  |  |  |  |
| BPRS: *Affective subscore* | **0.74****** | -0.29**** | -0.30**** | **0.52****** | 0.43**** |
| BPRS total | **0.58****** | -0.20**** | -0.22**** | 0.36**** | 0.33**** |
| BPRS: *Disorganization subscore* | 0.22**** |  |  |  |  |
| BPRS: *Negative subscore* |  |  |  |  |  |
| BPRS: *Positive subscore* |  |  |  |  |  |
| GF: Social | -0.26**** |  |  |  |  |
| GF: Role | -0.21**** |  |  |  |  |
| OASIS total | 0.45**** | -0.20**** | -0.20**** | 0.24**** |  |
| Perceived Stress Scale | **0.52****** | -0.21**** | -0.20**** | 0.32**** | 0.27**** |
| Perceived Discrimination total |  |  |  |  |  |
| PGI-S | 0.44**** |  | -0.20**** | 0.28**** | 0.25**** |
| PROMIS: Sleep quality score |  |  |  |  |  |
| PROMIS: Sleep disturbance score | 0.28**** |  |  | 0.20**** |  |
| C-SSRS: *Wish to be dead (lifetime)* | -0.28**** |  | **0.58****** | -0.35**** | -0.21**** |
| C-SSRS: *Non-specific suicidal thoughts (lifetime)* | -0.29**** | **0.58****** |  | -0.32**** | -0.33**** |
| C-SSRS: *Wish to be dead (past month)* | **0.50****** | -0.35**** | -0.32**** |  | **0.57****** |
| C-SSRS: *Non-specific suicidal thoughts (past month)* | 0.42**** | -0.21**** | -0.33**** | **0.57****** |  |
| p < 0.0001****  Abbreviations: PSYCHS, Positive Symptoms and Diagnostic Criteria for the CAARMS Harmonized with the SIPS; SIPS, Structured Interview for Psychosis Risk Syndromes; CAARMS, Comprehensive Assessment of the At-Risk Mental States; NSI-PR, Negative Symptom Inventory – Psychosis Risk; CDSS, Calgary Depression Scale for Schizophrenia; C-SSRS, Columbia-Suicide Severity Rating Scale; BPRS, Brief Psychiatric Rating Scale; GF: Social, Global Functioning: Social Scale; GF: Role, Global Functioning: Role Scale; PGI-S, Patient Global Impression of Severity; OASIS, Overall Anxiety Severity and Impairment Scale; PROMIS, Patient Reported Outcomes Measurement Information System. | | | | | |

| **Supplementary Table 3b: Correlation of Clinical Measures with Negative Symptoms & Functioning Measures** | | | | | | | |
| --- | --- | --- | --- | --- | --- | --- | --- |
|  | **NSI-PR: *Anhedonia*** | **NSI-PR: *Asociality*** | **NSI-PR: *Avolition*** | **NSI-PR: *Blunted affect*** | **NSI-PR: *Alogia*** | **GF: Social** | **GF: Role** |
| CDSS total | 0.28**** | 0.23**** | 0.34**** |  |  | -0.26**** | -0.21**** |
| NSI-PR: *Anhedonia* |  | 0.44**** | 0.45**** | 0.27**** |  | -0.30**** | -0.22**** |
| NSI-PR: *Asociality* | 0.44**** |  | 0.45**** | 0.33**** | 0.24**** | **-0.56****** | -0.29**** |
| NSI-PR: *Avolition* | 0.45**** | 0.45**** |  | 0.26**** | 0.20**** | -0.37**** | -0.49**** |
| NSI-PR: *Blunted affect* | 0.27**** | 0.33**** | 0.26**** |  | **0.60****** | -0.29**** |  |
| NSI-PR: *Alogia* |  | 0.24**** | 0.20**** | **0.60****** |  | -0.20**** |  |
| BPRS: *Activation subscore* |  |  |  |  |  |  | - |
| BPRS: *Affective subscore* | 0.23**** |  | 0.30**** |  |  | -0.22**** |  |
| BPRS total | 0.25**** | 0.29**** | 0.36**** | 0.25**** |  | -0.34**** | -0.28**** |
| BPRS: *Disorganization subscore* |  | 0.20**** | 0.29**** |  |  | -0.21**** | -0.24**** |
| BPRS: *Negative subscore* | 0.23**** | 0.24**** | 0.23**** | **0.69****** | 0.48**** | -0.28**** |  |
| BPRS: *Positive subscore* |  |  |  |  |  |  |  |
| GF: Social | -0.30**** | **-0.56****** | -0.37**** | -0.29**** | -0.20**** |  | 0.45**** |
| GF: Role | -0.22**** | -0.29**** | -0.49**** |  |  | 0.45**** |  |
| OASIS total |  | 0.21**** | 0.26**** |  |  |  |  |
| Perceived Stress Scale | 0.25**** |  | 0.32**** |  |  | -0.22**** | -0.21**** |
| Perceived Discrimination total |  |  |  |  |  |  |  |
| PGI-S |  |  | 0.25**** |  |  | -0.20**** | -0.20**** |
| PROMIS: Sleep quality score |  |  |  |  |  |  |  |
| PROMIS: Sleep disturbance score |  |  |  |  |  |  |  |
| C-SSRS: *Wish to be dead (lifetime)* |  |  |  |  |  |  |  |
| C-SSRS: *Non-specific suicidal thoughts (lifetime)* |  |  |  |  |  |  |  |
| C-SSRS: *Wish to be dead (past month)* |  |  | 0.22**** |  |  |  |  |
| C-SSRS: *Non-specific suicidal thoughts (past month)* |  |  |  |  |  |  |  |
| p < 0.0001.****  Abbreviations: PSYCHS, Positive Symptoms and Diagnostic Criteria for the CAARMS Harmonized with the SIPS; SIPS, Structured Interview for Psychosis Risk Syndromes; CAARMS, Comprehensive Assessment of the At-Risk Mental States; NSI-PR, Negative Symptom Inventory – Psychosis Risk; CDSS, Calgary Depression Scale for Schizophrenia; C-SSRS, Columbia-Suicide Severity Rating Scale; BPRS, Brief Psychiatric Rating Scale; GF: Social, Global Functioning: Social Scale; GF: Role, Global Functioning: Role Scale; PGI-S, Patient Global Impression of Severity; OASIS, Overall Anxiety Severity and Impairment Scale; PROMIS, Patient Reported Outcomes Measurement Information System. | | | | | | | |

| **Supplementary Table 3c: Correlation of Clinical Measures with BPRS** | | | | | | |
| --- | --- | --- | --- | --- | --- | --- |
|  | **BPRS: *Activation subscore*** | **BPRS: *Affective subscore*** | **BPRS total** | **BPRS: *Disorganization subscore*** | **BPRS: *Negative subscore*** | **BPRS: *Positive subscore*** |
| CDSS total |  | **0.74****** | **0.58****** | 0.22**** |  | 0.22**** |
| NSI-PR: *Anhedonia* |  |  | 0.25**** |  | 0.23**** |  |
| NSI-PR: *Asociality* |  |  | 0.29**** | 0.20**** | 0.24**** |  |
| NSI-PR: *Avolition* |  | 0.30**** | 0.36**** | 0.29**** | 0.23**** |  |
| NSI-PR: *Blunted affect* |  |  | 0.25**** |  | **0.69****** |  |
| NSI-PR: *Alogia* |  |  |  |  | 0.48**** |  |
| BPRS: *Activation subscore* |  |  |  |  |  |  |
| BPRS: *Affective subscore* |  |  | **0.68****** | 0.22**** |  | 0.24**** |
| BPRS total | 0.33**** | **0.68****** |  | 0.48**** | 0.34**** | **0.60****** |
| BPRS: *Disorganization subscore* |  | 0.22**** | 0.48**** |  |  |  |
| BPRS: *Negative subscore* |  |  |  |  |  |  |
| BPRS: *Positive subscore* | 0.21**** | 0.24**** | **0.60****** |  |  |  |
| GF: Social |  | -0.22**** | -0.34**** | -0.21**** | -0.28**** |  |
| GF: Role |  |  | -0.28**** | -0.24**** |  |  |
| OASIS total |  | 0.42**** | 0.40**** |  |  | 0.23**** |
| Perceived Stress Scale |  | 0.49**** | 0.45**** | 0.20**** |  |  |
| Perceived Discrimination total |  |  |  |  |  |  |
| PGI-S |  | 0.44**** | 0.38**** |  |  |  |
| PROMIS: Sleep quality score |  |  |  |  |  |  |
| PROMIS: Sleep disturbance score |  | 0.26**** | 0.24**** |  |  |  |
| C-SSRS: *Wish to be dead (lifetime)* |  | -0.29**** | -0.20**** |  |  |  |
| C-SSRS: *Non-specific suicidal thoughts (lifetime)* |  | -0.30**** | -0.22**** |  |  |  |
| C-SSRS: *Wish to be dead (past month)* |  | **0.52****** | 0.36**** |  |  |  |
| C-SSRS: *Non-specific suicidal thoughts (past month)* |  | 0.43**** | 0.33**** |  |  |  |
| p < 0.0001.****  Abbreviations: PSYCHS, Positive Symptoms and Diagnostic Criteria for the CAARMS Harmonized with the SIPS; SIPS, Structured Interview for Psychosis Risk Syndromes; CAARMS, Comprehensive Assessment of the At-Risk Mental States; NSI-PR, Negative Symptom Inventory – Psychosis Risk; CDSS, Calgary Depression Scale for Schizophrenia; C-SSRS, Columbia-Suicide Severity Rating Scale; BPRS, Brief Psychiatric Rating Scale; GF: Social, Global Functioning: Social Scale; GF: Role, Global Functioning: Role Scale; PGI-S, Patient Global Impression of Severity; OASIS, Overall Anxiety Severity and Impairment Scale; PROMIS, Patient Reported Outcomes Measurement Information System. | | | | | | |

| **Supplementary Table 3d: Correlation of Clinical Measures with Patient Reported Outcomes** | | | | | | |
| --- | --- | --- | --- | --- | --- | --- |
|  | **OASIS total** | **Perceived Stress Scale** | **Perceived Discrimination total** | **PGI-S** | **PROMIS: Sleep quality score** | **PROMIS: Sleep disturbance score** |
| CDSS total | 0.45**** | **0.52****** |  | 0.44**** |  | 0.28**** |
| NSI-PR: *Anhedonia* |  | 0.25**** |  |  |  |  |
| NSI-PR: *Asociality* | 0.21**** |  |  |  |  |  |
| NSI-PR: *Avolition* | 0.26**** | 0.32**** |  | 0.25**** |  |  |
| NSI-PR: *Blunted affect* |  |  |  |  |  |  |
| NSI-PR: *Alogia* |  |  |  |  |  | - |
| BPRS: *Activation subscore* |  |  |  |  |  |  |
| BPRS: *Affective subscore* | 0.42**** | 0.49**** |  | 0.44**** |  | 0.26**** |
| BPRS total | 0.40**** | 0.45**** |  | 0.38**** |  | 0.24**** |
| BPRS: *Disorganization subscore* |  | 0.20**** |  |  |  |  |
| BPRS: *Negative subscore* |  |  |  |  |  |  |
| BPRS: *Positive subscore* | 0.23**** |  |  |  |  |  |
| GF: Social |  | -0.22**** |  | -0.20**** |  |  |
| GF: Role | - | -0.21**** |  | -0.20**** |  |  |
| OASIS total |  | **0.63****** |  | **0.55****** |  | 0.33**** |
| Perceived Stress Scale | **0.63****** |  |  | **0.52****** |  | 0.33**** |
| Perceived Discrimination total |  |  |  |  |  |  |
| PGI-S | **0.55****** | **0.52****** |  |  |  | 0.30**** |
| PROMIS: Sleep quality score |  |  |  |  |  | -0.26**** |
| PROMIS: Sleep disturbance score | 0.33**** | 0.33**** |  | 0.30**** | -0.26**** |  |
| C-SSRS: *Wish to be dead (lifetime)* | -0.20**** | -0.21**** |  |  |  |  |
| C-SSRS: *Non-specific suicidal thoughts (lifetime)* | -0.20**** | -0.20**** |  | -0.20**** |  |  |
| C-SSRS: *Wish to be dead (past month)* | 0.24**** | 0.32**** |  | 0.28**** |  | 0.20**** |
| C-SSRS: *Non-specific*  *suicidal thoughts (past month)* |  | 0.27**** |  | 0.25**** |  |  |
| p < 0.05. * p < 0.01. ** p < 0.001. *** p < 0.0001.****  Abbreviations: PSYCHS, Positive Symptoms and Diagnostic Criteria for the CAARMS Harmonized with the SIPS; SIPS, Structured Interview for Psychosis Risk Syndromes; CAARMS, Comprehensive Assessment of the At-Risk Mental States; NSI-PR, Negative Symptom Inventory – Psychosis Risk; CDSS, Calgary Depression Scale for Schizophrenia; C-SSRS, Columbia-Suicide Severity Rating Scale; BPRS, Brief Psychiatric Rating Scale; GF: Social, Global Functioning: Social Scale; GF: Role, Global Functioning: Role Scale; PGI-S, Patient Global Impression of Severity; OASIS, Overall Anxiety Severity and Impairment Scale; PROMIS, Patient Reported Outcomes Measurement Information System. | | | | | | |
